# Analysis of autophagy deficiency and cytotoxicity in autophagy-deficient human embryonic stem cell-derived neurons

**DOI:** 10.1016/j.xpro.2023.102529

**Published:** 2023-08-25

**Authors:** Miriam E. Korsgen, Congxin Sun, Elena Seranova, Malgorzata Zatyka, Dewi Astuti, Tetsushi Kataura, Timothy Barrett, Viktor I. Korolchuk, Sovan Sarkar

**Affiliations:** 1 Institute of Cancer and Genomic Sciences, Institute of Biomedical Research, College of Medical and Dental Sciences, University of Birmingham, Edgbaston, Birmingham B15 2TT, UK; 2 Biosciences Institute, Faculty of Medical Sciences, Newcastle University, Newcastle upon Tyne NE4 5PL, UK; 3 Department of Endocrinology, Birmingham Women’s and Children’s Hospital, Steelhouse Lane, Birmingham B4 6NH, UK

**Keywords:** Cell Biology, Cell Culture, Cell-Based Assays, Stem Cells, Cell Differentiation

## Abstract

Autophagy, a catabolic process governing cellular and energy homeostasis, is essential for cell survival and human health. Here, we present a protocol for generating autophagy-deficient (*ATG5*^*−/−*^) human neurons from human embryonic stem cell (hESC)-derived neural precursors. We describe steps for analyzing loss of autophagy by immunoblotting. We then detail analysis of cell death by luminescence-based cytotoxicity assay and fluorescence-based TUNEL staining. This hESC-based experimental platform provides a genetic knockout model for undertaking autophagy studies relevant to human biology.

For complete details on the use and execution of this protocol, please refer to Sun et al. (2023).^1^

## Highlights

• Protocol for differentiation of hESC-derived neural precursors into neurons
• Characterization of autophagy deficiency through immunoblot analysis of LC3 and p62
• Analysis of cell death in hESC-derived neurons by cytotoxicity and TUNEL assays
• Loss of autophagy causes cell death in *ATG5*^*−/−*^ hESC-derived neurons

## Abstract (editor highlights)

Publisher’s note: Undertaking any experimental protocol requires adherence to local institutional guidelines for laboratory safety and ethics.

## Graphical abstract

**Figure:**
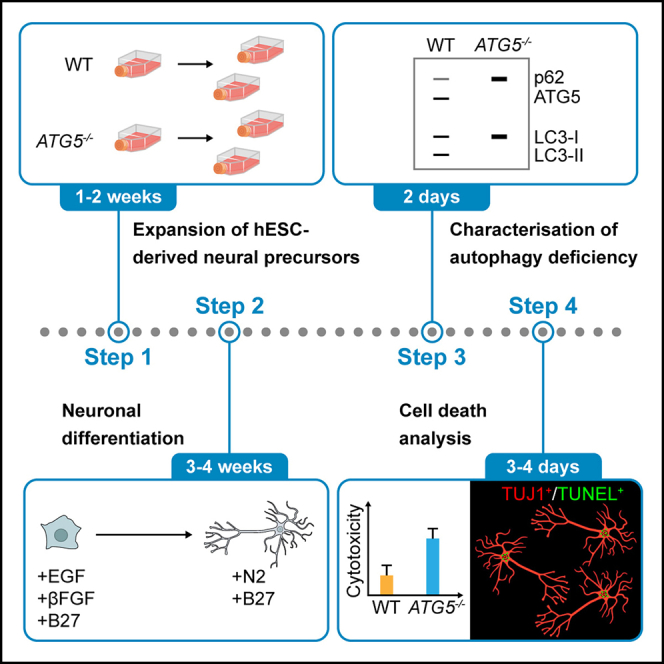

## Before you begin

### Institutional permissions

The users need to get approval from their relevant institutions confirming that all experiments conform to the relevant regulatory and safety standards. The users also need to acquire permission from their institution’s ethical committee for the use of human pluripotent stem cell models for research in accordance with relevant institutional and national guidelines and regulations.

### Preparation of tissue culture plates and media

**Timing: 1 day**

The human embryonic stem cell (hESC) lines used in this protocol are wild-type (*ATG5*^*+/+*^)^2^ and autophagy-deficient (*ATG5*^*−/−*^).^1^ The autophagy-deficient hESC lines (*ATG5*^*−/−*^) were generated from the parental WIBR3 hESC line (*ATG5*^*+/+*^) by knockout of exon 3 of an essential autophagy gene, *ATG5*, by genome editing with transcription activator-like effector nucleases (TALENs).^1^ The hESC lines were cultured feeder-free on Geltrex or Matrigel basement membrane matrix prior to differentiation. Detailed methods on the generation and characterization of these hESC lines and their differentiation into neural precursors (NPs) can be found in our original paper.^1^ The NPs were generated from hESCs via the ‘dual SMAD inhibition’ method previously described by Chambers and colleagues,^3^ but with some modifications as we have recently reported.^1^ While the previous protocol demonstrated highly efficient neural conversion of human pluripotent stem cells through monolayer cultures,^3^ we have incorporated the use of three-dimensional neurospheres along with a short-term application of dual-SMAD inhibition.^1^^,^^4^ The hESC-derived NPs are cultured and differentiated into neurons on poly-L-ornithine and laminin (PO-L) coated plates,^1^ as described in this protocol.

1. 24 h before you plan to thaw or seed cells, prepare PO-L coated plates.
  a. Coat culture plates or flasks with 100 μg/mL poly-L-ornithine in Dulbecco’s phosphate-buffered saline (DPBS), and incubate for 2 h at 37°C.
  b. Remove poly-L-ornithine solution and wash 3 times with DPBS.
  c. Coat plates with laminin in DPBS at 1–2 μg/cm^2^ and incubate overnight at 37°C.

***Note:*** When first establishing the NPs in culture we recommend starting in a 6-well plate before scaling up to ensure high density culture.

2. Prepare 500 mL NP culture medium without epidermal growth factor (EGF) and fibroblast growth factor 2 (FGF-2). Complete NP culture medium by supplementing with growth factors is made in small quantities (e.g., 40 mL) immediately before use to ensure fresh supply of growth factors to cells.

## Key resources table

**Table:** 

REAGENT or RESOURCE	SOURCE	IDENTIFIER
**Antibodies**		

ATG5 monoclonal antibody (mouse); 1:400 dilution for immunoblotting	NanoTools	0262-100/ATG5-7C6
GAPDH monoclonal antibody (mouse); 1:5000 dilution for immunoblotting	Sigma-Aldrich	Cat# G8795; RRID: AB_1078991
LC3B polyclonal antibody (rabbit); 1:2000 dilution for immunoblotting	Novus Biologicals	Cat# NB100-2220; RRID: AB_10003146
p62 monoclonal antibody (mouse); 1:1000 dilution for immunoblotting	BD Biosciences	Cat# 610832; RRID: AB_398151
TUJ1 (TUBB3) monoclonal antibody (mouse); 1:200 dilution for immunofluorescence	BioLegend	Cat# 801201; RRID: AB_2313773
Goat anti-mouse IgG, H&L chain specific peroxidase conjugate; 1:10000 dilution for immunoblotting	Calbiochem	Cat# 401253; RRID: AB_437779
Goat anti-rabbit IgG, H&L chain specific peroxidase conjugate; 1:10000 dilution for immunoblotting	Calbiochem	Cat# 401393; RRID: AB_437797
Goat anti-mouse IgG (H + L), Alexa Fluor 594; 1:1000 dilution for immunofluorescence	Invitrogen	Cat# A-11005; RRID: AB_2534073

**Chemicals, peptides, and recombinant proteins**		

2-mercaptoethanol	Gibco	Cat# 31350010
Amersham ECL Western Blotting Detection Reagent	Cytiva	Cat# RPN2106
Animal-free recombinant human EGF	PeproTech	Cat# AF-100-15
B-27 Supplement (50×), minus vitamin A	Gibco	Cat# 12587010
Bovine serum albumin	Sigma-Aldrich	Cat# A7030
Complete Mini Protease Inhibitor Cocktail tablets	Roche	Cat# 11836153001
Deionized water (DNase/RNase-free)	Promega	Cat# P119C
Dimethyl sulfoxide (DMSO)	Sigma-Aldrich	Cat# D2650
DMEM/F-12	Gibco	Cat# 11320033
DPBS (without calcium and magnesium)	Gibco	Cat# 14190094
Glycine	Sigma-Aldrich	Cat# G8898
Human FGF-2, premium grade	Miltenyi Biotec	Cat# 130-093-842
KnockOut Serum Replacement	Gibco	Cat# 10828010
Laminin	Sigma-Aldrich	Cat# L2020
L-glutamine (200 mM)	Gibco	Cat# 25030024
MEM non-essential amino acids solution (100×)	Gibco	Cat# 11140035
Methanol	VWR Chemicals	Cat# 20847
N-2 supplement (100×)	Gibco	Cat# 17502048
Paraformaldehyde (16%)	Thermo Fisher Scientific	Cat# 28908
PBS tablets	Gibco	Cat# 18912014
Penicillin-Streptomycin (5000 U/mL)	Gibco	Cat# 15070063
Poly-L-ornithine	Sigma-Aldrich	Cat# P4957
ProLong Gold antifade reagent with DAPI	Invitrogen	Cat# P36931
RevitaCell Supplement (100×)	Gibco	Cat# A2644501
RIPA buffer	Sigma-Aldrich	Cat# R0278
Sodium dodecyl sulfate	Sigma-Aldrich	Cat# 75746
StemPro Accutase Cell Dissociation Reagent	Gibco	Cat# A1110501
Tris-HCl	Sigma-Aldrich	Cat# 1083150100
Triton X-100	Sigma-Aldrich	Cat# 648466
Trizma base	Sigma-Aldrich	Cat# T1503
Trypan blue stain (0.4%)	Gibco	Cat# 15250061
Trypsin-EDTA (0.05%), phenol red	Gibco	Cat# 25300054
Tween 20	Sigma-Aldrich	Cat# P7949

**Critical commercial assays**		

Bio-Rad Protein Assay Kit II	Bio-Rad	Cat# 5000002
CytoTox-Glo Cytotoxicity Assay	Promega	Cat# G9290
Click-iT Plus TUNEL assay for in situ apoptosis detection, Alexa Fluor 488 dye	Invitrogen	Cat# C10617

**Experimental models: Cell lines**		

*ATG5*^+/+^ (WIBR3) hESCs	Lengner et al.^2^	N/A
*ATG5*^−/−^ (clones #5) hESCs	Sun et al.^1^	N/A

**Software and algorithms**		

GraphPad Prism v8.3.1	GraphPad Software	https://www.graphpad.com/; RRID: SCR_002798
ImageJ v1.41	NIH	https://imagej.net/ij/index.html; RRID: SCR_003070

## Materials and equipment

**Table:** Neural precursor (NP) culture medium

Reagent	Final concentration	Amount
DMEM/F-12	N/A	469.8 mL
B-27 plus supplement (50×)	1×	10 mL
N-2 Supplement (100×)	1×	5 mL
L-glutamine (200 mM)	1% (v/v)	5 mL
Penicillin-Streptomycin (5000 U/mL)	50 U/mL	5 mL
MEM non-essential amino acids solution (100×)	1×	5 mL
Human FGF-2 (100 μg/mL)	20 ng/mL	100 μL
Animal-Free Recombinant Human EGF (100 μg/mL)	20 ng/mL	100 μL
**Total**	**N/A**	**500 mL**

NP culture medium is first made up to 500 mL without growth factors and stored at 4°C for up to 1 month. Complete NP media with EGF and FGF-2 can be stored at 4°C for up to 1 week.

**Table:** Neural precursor (NP) freezing medium

Reagent	Final concentration	Amount
KnockOut Serum Replacement	N/A	890 μL
Dimethyl sulfoxide	10% (v/v)	100 μL
RevitaCell supplement (100×)	1×	10 μL
**Total**	**N/A**	**1 mL**

Make fresh each time before using.

**Table:** Neuronal differentiation medium

Reagent	Final concentration	Amount
DMEM/F-12	N/A	470 mL
B-27 plus supplement (50×)	1×	10 mL
N-2 Supplement (100×)	1×	5 mL
L-glutamine (200 mM)	1% (v/v)	5 mL
Penicillin-Streptomycin (5000 U/mL)	50 U/mL	5 mL
MEM non-essential amino acids solution (100×)	1×	5 mL
**Total**	**N/A**	**500 mL**

Store at 4°C for up to 1 month.

**Table:** Lysis Buffer with Protease Inhibitor Cocktail (1×)

Reagent	Final concentration	Amount
RIPA Buffer	1×	10 mL
Complete Mini Protease Inhibitor Cocktail tablet	N/A	1 tablet
**Total**	**N/A**	**10 mL**

Store at −20°C for up to 3 month as frozen aliquots.

### Running Buffer (1×)

Make up 10× Running Buffer (Tris 250 mM, Glycine 1.92 M), dilute to 1× as needed.

**Table:** 

Reagent	Final concentration	Amount
Tris-Glycine Running Buffer (10×)	Tris 25 mM, Glycine 192 mM	100 mL
Sodium dodecyl sulfate (10%)	1% (v/v)	10 mL
ddH_2_O	N/A	890 mL
**Total**	**N/A**	**1 L**

Store at room temperature for up to 1 month.

### Transfer Buffer (1×)

Make up 10× Transfer Buffer (Tris 250 mM, Glycine 1.92 M), dilute to 1× as needed.

**Table:** 

Reagent	Final concentration	Amount
Tris-Glycine Transfer Buffer (10×)	Tris 25 mM, Glycine 192 mM	200 mL
Methanol	20% (v/v)	400 mL
ddH_2_O	N/A	1.4 L
**Total**	**N/A**	**2 L**

Store at room temperature for up to 1 month.

**Table:** PBS-T (1×)

Reagent	Final concentration	Amount
PBS tablet	1×	2 tablets
Tween 20	2% (v/v)	2 mL
ddH_2_O	N/A	Up to 1 L
**Total**	**N/A**	**1 L**

Store at room temperature for up to 3 months.

**Table:** Stripping Buffer (1×)

Reagent	Final concentration	Amount
Sodium dodecyl sulfate (10%)	2% (v/v)	20 mL
Tris-HCL (0.5 M, pH 6.8)	62.5 mM	12.5 mL
2-mercaptoethanol (50 mM)	0.4 mM	0.8 mL
ddH_2_O	N/A	66.7 mL
**Total**	**N/A**	**100 mL**

2-mercaptoethanol is hazardous, make buffer just before use in a fume hood. Store at room temperature for up to 1 week.

## Step-by-step method details

### Expansion of hESC-derived neural precursors

**Timing: 1–2 weeks**

The NPs generated from hESCs can be stored as frozen stocks that can be revived to be differentiated into neurons for experimentation. Terminally differentiated human neurons, generated from hESC-derived NPs, are post-mitotic and will not divide after the differentiation process.^4^ Therefore, the wild-type (*ATG5*^*+/+*^) and autophagy-deficient (*ATG5*^*−/−*^) hESC- derived NPs must be first expanded and then seeded at appropriate densities for neuronal differentiation as per the requirement of the experiments. NPs differentiated from multiple clones of genome-edited *ATG5*^*−/−*^ hESCs are recommended to avoid clonal effects. However, this is not necessary for NPs derived from the wild-type parental hESCs on which genome editing was performed to generate the autophagy-deficient clonal lines.

1. Establish *ATG5*^*+/+*^ and 3 independent clones of *ATG5*^*−/−*^ hESC-derived NPs in culture.
  a. Warm the pre-prepared NP culture medium without growth factors at 37°C in a water bath for at least 30 min for use in the steps below.
  b. Make up 40 mL of complete NP culture medium by adding 8 μL EGF (from 100 μg/mL stock) and 8 μL FGF-2 (from 100 μg/mL stock) to the pre-prepared NP culture medium without growth factors. Mix by inversion.
  c. Remove laminin solution from plates, add NP culture medium (1 mL per well of 6-well plate) and warm in the incubator at 37°C.
  d. Remove frozen NP vials from liquid nitrogen and rapidly thaw at 37°C in a water bath. Clean cryovial with ethanol, then immediately transfer cells by adding dropwise to a 15 mL Falcon tube containing 8 mL of NP culture medium.
  e. Spin cell suspension in a centrifuge at 200 × *g* for 3 min at room temperature.
  f. Remove supernatant completely, taking care not to disturb the cell pellet. Resuspend cells in 1 mL of NP culture medium.
  g. Take the PO-L coated warmed plate containing NP medium out of the incubator and add 1 mL cell suspension dropwise per well of 6-well plate. Move plate gently in crisscross motion to distribute cells evenly in the well.
  h. Place the plate in a humidified incubator at 37°C and do not disturb for at 48 h to allow the cells to attach. Repeat the process for each cell line.

**Figure 1:**
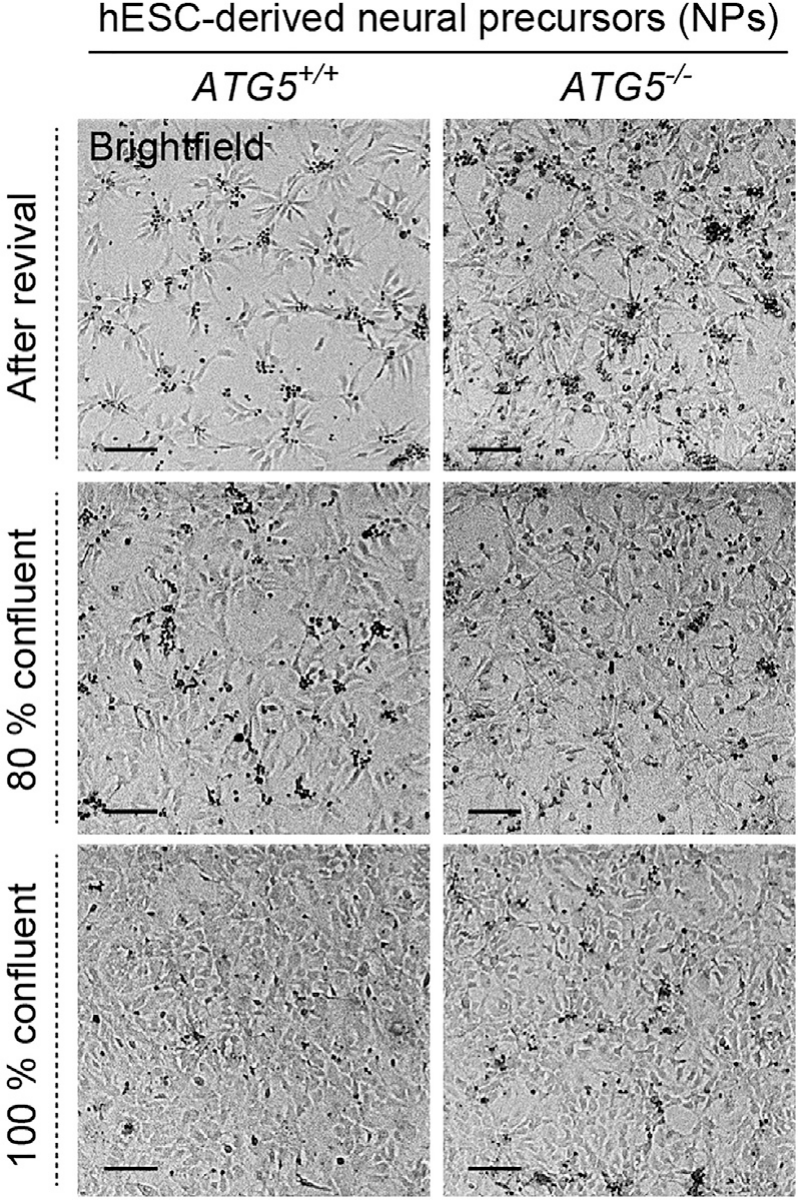
Morphology and density of hESC-derived neural precursors Representative brightfield microscopy images of *ATG5*^+/+^ (wild-type) and *ATG5*^*−/−*^ (autophagy-deficient; clone #5) hESC-derived neural precursors (NPs), cultured on poly-L-ornithine/laminin coated 6-well plates, after revival from frozen stock, at 80% confluency and at 100% confluency. Scale bar: 100 μm.

***Optional:*** When reviving frozen stocks of NPs, RevitaCell Supplement (1:100 dilution) can be added to the complete NP culture medium at step 1b to improve cell viability during post-thaw recovery.

2. Culture and freeze down NPs.
  a. Culture hESC-derived NPs in a humidified incubator with 5% CO_2_ at 37°C. Change media every day. When culturing NPs, media can be aspirated using a P1000 or vacuum pump system.
  b. Passage cells once they become 100% confluent.
    **CRITICAL:** Split NPs at 1:2 or 1:3 ratio, maintaining a high density to promote cell growth and reduce differentiation risk (Figure 1).
    i. Coat 6-well plates according to the first Preparation step at least 24 h before passaging cells.
    ii. Warm NP culture medium and DPBS at 37°C in a water bath for at least 10 min.
    iii. Remove laminin solution from PO-L coated plate, add NP culture medium (1 mL per well of 6-well plate) and warm the plate in incubator at 37°C.
    iv. Remove media from 6-well plate containing the NPs and wash cells once with DPBS.
    v. Remove DPBS and add Accutase dissociation reagent (1 mL per well of 6-well plate) dropwise to cover surface of well.
    vi. Incubate cells at 37°C for 3–5 min. Gently tap the plate afterwards to dissociate the cells.
    vii. Neutralize Accutase with 3 mL DPBS (1:3 ratio) per well.
    viii. Transfer cell suspension into a 15 mL Falcon tube. Tilt the plate and gently flush any remaining cells from the well with DPBS and collect in Falcon tube.
    ix. Spin cell suspension at 200 × *g* for 3 min at room temperature.
    x. Remove supernatant completely, taking care not to disturb the cell pellet.
    xi. Resuspend cells in 1 mL NP culture medium.
    xii. Take media-containing pre-warmed PO-L coated plate out of the incubator add 1 mL cell suspension dropwise per well. Move plate in a crisscross motion to distribute cells evenly.
    xiii. Place plate in a humidified incubator at 37°C and do not disturb for 24 h to allow the cells to attach.
      ***Optional:*** NPs can be counted at step 2bxi after resuspending cells in NP culture medium. This can be done to maintain the same density across the cultures of different cell lines. Also, NP cultures can be scaled up into T75 PO-L coated flasks. We experienced that Accutase Dissociation Reagent may not be effective in flasks. Below is an alternative dissociation protocol using 0.05% Trypsin-EDTA.
      **CRITICAL:** A minimum of 5 × 10^6^ cells is required to seed a T75 flask to ensure an appropriate cell density for preventing spontaneous differentiation and promoting cell growth.
  c. Alternative dissociation protocol for culture flasks using 0.05% Trypsin-EDTA.
    i. Coat T75 flasks according to the first Preparation step at least 24 h before passaging cells.
    ii. Warm NP culture medium, DPBS and KnockOut Serum Replacement (KOSR) at 37°C in a water bath for at least 10 min.
    iii. Remove laminin solution from PO-L coated flask, add 14 mL NP culture medium and warm the flask in incubator at 37°C.
    iv. Remove media from T75 flask containing the NPs and wash cells once with DPBS.
    v. Remove DPBS and add 2 mL of 0.05% Trypsin-EDTA to dissociate the cells.
    vi. Incubate cells at 37°C for 1–3 min. Gently tap the flask afterwards.
    vii. Quench Trypsin-EDTA with 1 mL KOSR, dilute further with 7 mL NP culture medium and transfer the cell suspension into a 15 mL Falcon tube.
    viii. Spin cell suspension at 200 × *g* for 3 min at 21°C.
    ix. Remove supernatant completely, taking care not to disturb the cell pellet.
    x. Resuspend cells in 1 mL NP culture medium.
    xi. Take NP medium-containing warmed PO-L coated flask out of the incubator and add 1 mL cell suspension to it. Move flask in a crisscross motion to distribute the cells evenly.
    xii. Place flask in a humidified incubator at 37°C and do not disturb for 24 h to allow the cells to attach.
  d. To freeze down NPs, follow dissociation protocol in steps 2biv–x or 2civ–ix without preparing a culture plate or flask.
    i. Prepare NP freezing medium (1 mL per cryovial) and keep inside the hood at room temperature for at least 30 min.
    ii. Resuspend cells in 1 mL NP freezing medium and transfer all the cell suspension into a labelled cryovial.
    iii. Place cryovial into a freezing chamber at −80°C freezer before transferring to liquid nitrogen 24 h later.
      **CRITICAL:** Freeze down hESC-derived NPs of each cell line regularly to maintain backup of frozen stocks in case of spontaneous differentiation or contamination during NP culture.
      ***Optional:*** The cellular identity of hESC-derived NPs can be confirmed by lineage-specific markers, such as NESTIN and PAX6,^5^ through immunofluorescence, immunoblotting or qPCR.^1^

### Terminal differentiation of hESC-derived neural precursors into neurons

**Timing: 3 weeks**

The hESC-derived NPs, generated via the dual SMAD inhibition method,^3^ possess an anterior identity and commit to forebrain fates upon culturing in the presence of bFGF and EGF.^5^ Terminally differentiated human neurons are generated from hESC-derived NPs after growth factor withdrawal. These neuronal cells are post-mitotic and will not divide after the differentiation process.^4^ Therefore, *ATG5*^*+/+*^ and *ATG5*^*−/−*^ hESC-derived NPs are seeded at appropriate densities for neuronal differentiation as per the requirement of the experiments. Since loss of autophagy contributes to neurotoxicity,^6^^,^^7^^,^^8^^,^^9^ substantial cell death is observed in *ATG5*^*−/−*^ hESC-derived neurons after 3 weeks of neuronal differentiation.^1^ Therefore, experiments can be undertaken in 3-weeks old neurons, although the neuronal cultures can be maintained up to 4–5 weeks.^1^

**Figure 2:**
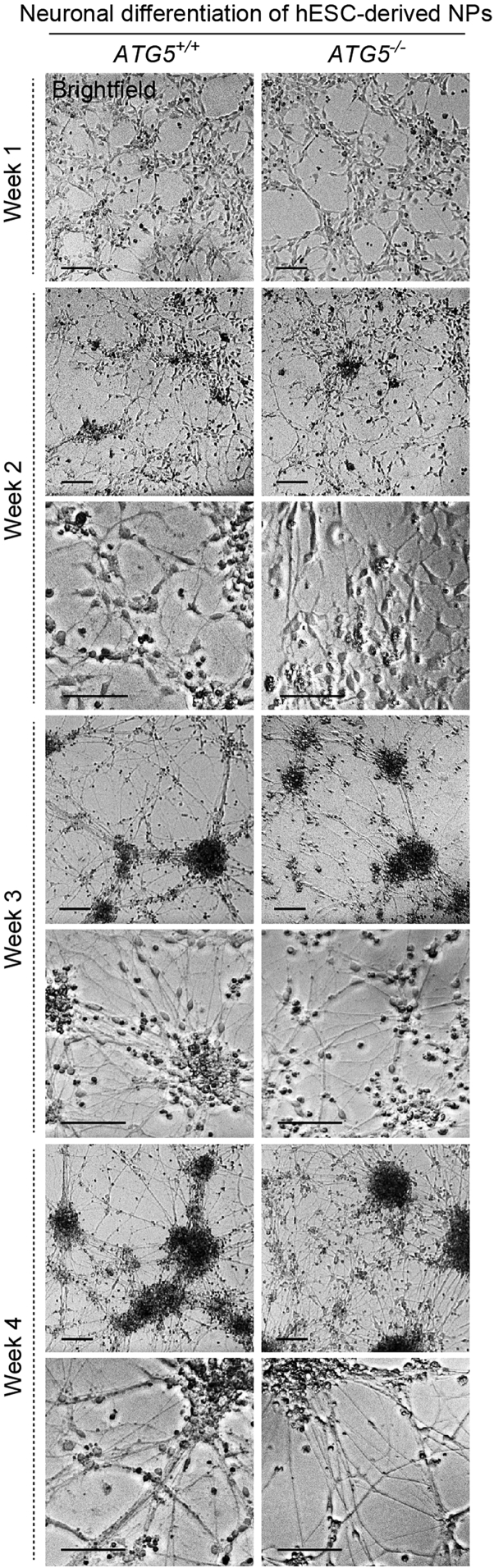
Morphology of hESC-derived cells during neuronal differentiation Representative brightfield microscopy images of *ATG5*^+/+^ (wild-type) and *ATG5*^*−/−*^ (autophagy-deficient; clone #5). hESC-derived cells, cultured on poly-L-ornithine/laminin coated 6-well plates, after 1, 2, 3 and 4 weeks of neuronal differentiation from hESC-derived neural precursors (NPs). Scale bar: 100 μm.

3. Differentiate hESC-derived NPs into neurons.
  a. Dissociate the NPs in 6-well plate according to steps 2biv–x (or steps 2civ–xi if using flasks) and seed NPs into appropriate PO-L coated plates for experiments.
    **CRITICAL:** At this stage, instead of using NP culture medium, switch to neuronal differentiation medium (without EGF and FGF-2) containing N-2 and B-27 supplements to resuspend the NP pellets. Seeding densities shown in Table 1.
    i. Coat experimental plates, with or without coverslips as necessary, according to the first Preparation step at least 24 h before passaging cells.
    ii. Warm neuronal differentiation medium at 37°C in a water bath for at least 30 min.
    iii. Remove laminin solution from PO-L coated plate, add neuronal culture medium (1 mL, 500 μL or 100 μL per well of 6-, 24- or 96-well plate, respectively) and warm the plate in incubator at 37°C.
    iv. From steps 2bx or 2cix, resuspend NP cell pellet in 1 mL neuronal differentiation medium.
    v. Count cells using 10 μL cell suspension and Trypan Blue staining. Adjust cell suspension volume with neuronal differentiation medium as per Table 1 in order to dropwise add 1 mL, 500 μL or 100 μL of cell suspension per well of 6-, 24- or 96-well plate, respectively.
    vi. Seed one 6-well plate per NP line (wild-type line and independent clonal lines of *ATG5*^*−/−*^) for immunoblotting analysis.
    vii. Seed a 24-well plate containing glass coverslips with at least 3 replicates of each cell line for immunofluorescence and TUNEL staining.
    viii. Seed a 96-well plate containing at least 6 replicates of each cell line, for the cytotoxicity assay.
    ix. Place the plates in a humidified incubator at 37°C and do not disturb for 24 h to allow the cells to attach.
  b. Culture cells in neuronal differentiation medium for 3–4 weeks (Figure 2).
    i. Change medium every alternate day using a P1000 pipette (for 6-well or 24- well plate) or a multichannel pipette (for 96-well plate). Tilt the plate and slowly remove 70% of the media from each well of the plate to avoid disrupting the neurons. Replenish with fresh, pre-warmed neuronal differentiation medium slowly and gently.
      ***Note:*** This protocol produces a mixed population of terminally differentiated neurons.^4^^,^^10^^,^^11^ The cells become more delicate as neuronal differentiation progresses.
      ***Optional:*** Neuronal identity can be confirmed by neuron-specific markers such as MAP2 and TUJ1,^4^^,^^5^ through immunofluorescence, immunoblotting or qPCR.^1^

**Table 1:** Seeding densities for differentiation

Culture plate/flask	Surface area per well	Neural precursors per well
6-well	9.6 cm^2^	8 × 10^5^ cells
24-well	1.9 cm^2^	1.8 × 10^5^ cells
96-well	0.32 cm^2^	8 × 10^4^ cells

### Characterization of autophagy deficiency by immunoblotting

**Timing: 3 days**

**Figure 3:**
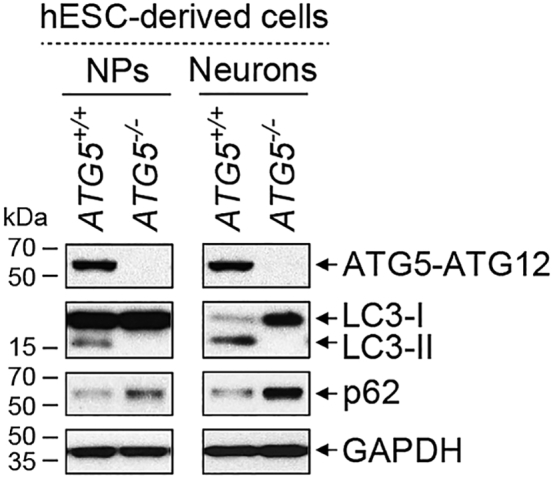
Immunoblotting analysis of autophagy deficiency in hESC-derived NPs and neurons Immunoblotting analysis of ATG5, LC3B, p62 and GAPDH in *ATG5*^+/+^ (wild-type) and *ATG5*^*−/−*^ (autophagy-deficient; clone #5) hESC-derived NPs (left panel) and neurons after 4 weeks of neuronal differentiation (right panel).

To analyze autophagy deficiency, immunoblotting analysis is done to compare the levels of ATG5 (the product of the essential autophagy gene that is deleted),^12^^,^^13^ LC3B-II (autophagosomes),^14^^,^^15^^,^^16^ and p62 (autophagy substrate)^17^ between wild-type (*ATG5*^*+/+*^) and autophagy-deficient (*ATG5*^*−/−*^) hESC-derived NPs and neurons. *ATG5*^*−/−*^ cells will exhibit absence of ATG5 and LC3B-II, and accumulation of p62, indicating loss of autophagy^1^ (Figure 3). The hESC-derived NPs are seeded into 6-well plates, and either harvested upon confluency for immunoblotting or differentiated for 3–4 weeks into neurons according to step 3. Neurons are harvested for immunoblotting after 3–4 weeks.^18^

4. Harvest cells and prepare lysate for immunoblotting.
  a. Remove medium and wash each well gently with 1 mL DPBS, then remove slowly.
  b. Use 1 mL DPBS per well to harvest cells, scrape to detach cells using a cell scraper and collect cell suspension. For each cell line, pool 2 wells into 1 Eppendorf tube, creating n = 3 for each cell line from one 6-well plate. Keep cell suspension on ice.
  c. Centrifuge cell suspension at 2400 × *g* at 4°C for 5 min.
  d. Discard supernatant and keep cell pellets on ice.
    **Pause point:** Once collected, cell pellets can be stored at −80°C freezer for up to 3–6 months.
  e. Depending on size of cell pellet, resuspend in appropriate volume (such as 50–100 μL per pellet) of Lysis Buffer with protease inhibitor cocktail.
  f. Sonicate the cell lysates at 9 kHz for 10 s, repeat 3 times.
  g. Vortex cell lysates for 5 s and incubate on ice for 30 min.
  h. Centrifuge cell lysates at 9600 × *g* at 4°C for 5 min.
  i. Collect supernatant (cell lysate) of each sample in new Eppendorf tube and keep on ice, discard the pellets (cell debris).
    **Pause point:** Once prepared, cell lysates can be stored at −80°C freezer for up to 3–6 months.
  j. Quantify protein concentration in cell lysate using Bio-Rad Protein Assay Kit II according to manufacturer’s instructions.
  k. Prepare to load 20 μg protein per cell lysate. For example, adjust loading volume of cell lysate by making up to 10–20 μL using Lysis Buffer containing protease inhibitor cocktail and then add 2–4 μL of 6× SDS loading dye.
  l. Vortex the cell lysate dye mixture for 5 s and boil samples at 95°C for 5 min on a heating block.
5. Perform immunoblotting analysis.
  a. Load appropriate volume of the cell lysate dye mixture containing 20 μg of protein (such as 12–24 μL for each sample) along with 10 μL of a protein ladder into a 12% (w/v) precast polyacrylamide gel.
  b. Perform SDS-PAGE (sodium dodecyl sulfate polyacrylamide gel electrophoresis) using 1× Running Buffer under 120 V for 2–2.5 h until the smallest molecular weight marker reaches the bottom of the gel (∼10 kDa).
  c. Transfer the protein from polyacrylamide gel on to methanol-activated PVDF membrane under 90 V for 90 min on ice using 1× Transfer Buffer with methanol.
  d. Block PVDF membrane in 5% (w/v) skimmed milk in PBS-T (phosphate-buffered saline with Tween 20) for 1 h at room temperature.
    ***Note:*** The membrane is cut into 3 portions at appropriate locations, using the protein ladder as a guide, to probe for ATG5 and p62 (top), GAPDH (middle) and LC3B (for LC3B-I and LC3B-II; bottom).
  e. Incubate each membrane with respective primary antibodies for ATG5, LC3B and GAPDH (loading control) on a rocking platform overnight at 4°C.
  f. Wash membranes 6 times with PBS-T for 10 min each time, on a rocking platform at room temperature.
  g. Incubate each membrane with appropriate HRP-conjugated secondary antibodies on a rocking platform for 1 h at room temperature.
  h. Wash membranes 6 times with PBS-T for 10 min each time, on a rocking platform at room temperature.
  i. Add ECL Western Blotting Detection Reagent to each membrane for 1 min at room temperature, followed by exposing and developing on autoradiography film to visualize signal.
  j. Strip top part of the membrane with Stripping Buffer at 65°C for 15 min.
  k. Block membrane as in step 5d, then incubate with primary antibody for p62 on a rocking platform overnight at 4°C, followed by steps 5f–i.
    ***Note:*** Expected band size is 62 kDa for p62, 55 kDa for ATG5 (which is normally detected as a conjugation product with ATG12), 37 kDa for GAPDH, 18 kDa for LC3B-I and 16 kDa for LC3B-II (Figure 3).
    ***Optional:*** Autophagy deficiency can also be characterized by loss of LC3B and WIPI2 puncta via immunofluorescent analysis.^12^^,^^14^^,^^19^ We have demonstrated that *ATG5*^*−/−*^ hESCs and hESC-derived NPs and neurons do not form LC3B puncta in our original paper.^1^

### Cell death analysis in autophagy-deficient neurons by cytotoxicity assay

**Timing: 2–3 h**

**Figure 4:**
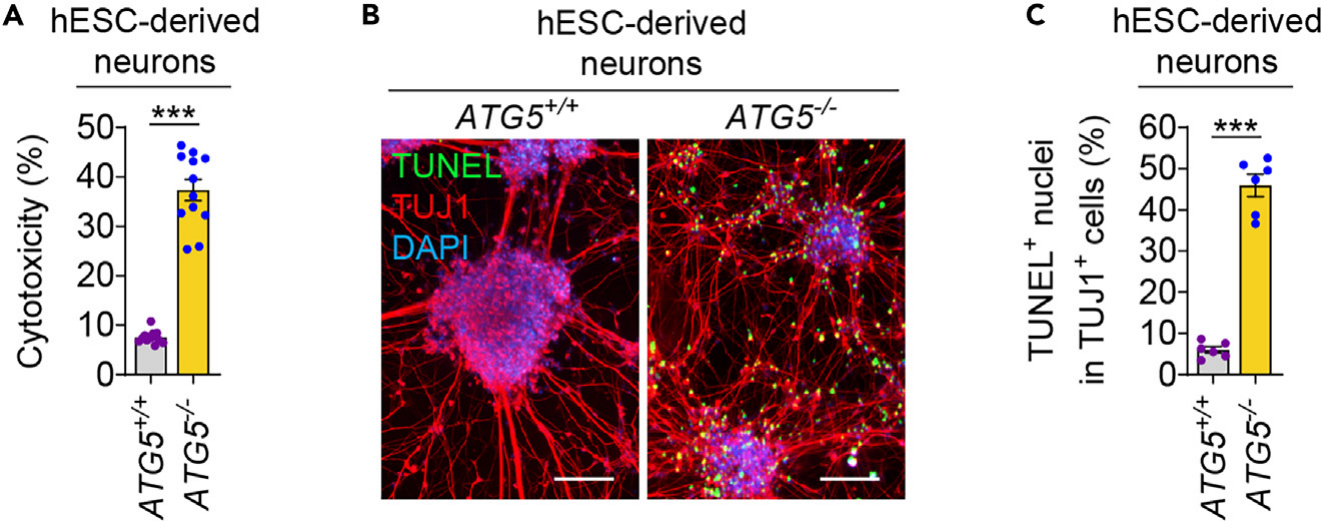
Analysis of cell death due to autophagy deficiency in hESC-derived neurons (A–C) Cytotoxicity analysis (A), immunofluorescence images of TUJ1 with TUNEL staining (B), and quantification of TUNEL^+^ apoptotic nuclei (C) in *ATG5*^+/+^ (wild-type) and *ATG5*^*−/−*^ (autophagy-deficient; clone #5) hESC-derived neurons after 3 weeks of neuronal differentiation. Graphical data are represented as mean ± s.e.m. of *n* = 6 (C) or 12 (A) biological replicates. *P* values were calculated by unpaired two-tailed Student’s *t*-test on 3 independent experiments (A, C). ∗∗∗*P* < 0.001. Scale bar: 100 μm (B).

Autophagy is essential for maintaining cellular homeostasis, whereas loss of autophagy is detrimental to cellular survival and contributes to cytotoxicity.^1^^,^^6^^,^^7^^,^^8^^,^^9^^,^^12^^,^^20^ Increased cell death is observed in *ATG5*^*−/−*^ hESCs and hESC-derived NPs, and this phenotype is aggravated as the neuronal differentiation progresses.^1^ To evaluate the impact of autophagy deficiency on neuronal viability at a population level, cytotoxicity analysis is performed using CytoTox-Glo Cytotoxicity Assay (Promega).^21^ This luminescence-based cytotoxicity assay measures the extracellular activity of a dead-cell protease after being released from membrane-compromised cells. The hESC-derived NPs are seeded into 96-well PO-L coated plates and then differentiated into neurons according to step 3. Cytotoxicity is measured after 3 weeks of neuronal differentiation where considerable cell death is observed in *ATG5*^*−/−*^ hESC- derived neurons^1^ (Figure 4A).

6. Perform using CytoTox-Glo Cytotoxicity Assay (Promega) on hESC-derived neurons, according to manufacturer’s instructions.
  a. Measure basal cytotoxicity from dead cells in the population (first reading).
    i. Prepare AAF-Glo Assay Reagent in Promega Kit from 5 mL Assay Buffer and AAF-Glo Substrate powder.
    ii. Gently remove old media and slowly add 100 μL fresh neuronal differentiation medium to each well of 96-well plate. Avoid harsh pipetting as this will detach the neuronal network from the bottom of the well.
    iii. Gently add 50 μL AAF-Glo solution to each well of 96-well plate and slowly pipette up and down twice to mix.
    iv. Cover the plate with foil and incubate for 15 min at room temperature.
    v. Measure luminescence with a microplate reader, take two readings to average.
  b. Measure induced cytotoxicity from total cells in the population (second reading).
    i. Prepare Lysis Reagent in Promega Kit from 5 mL Assay Buffer and 33 μL Digitonin.
    ii. To the same plate, gently add 50 μL Lysis Reagent to each well of 96-well plate and slowly pipette up and down twice to mix.
    iii. Cover the plate with foil and incubate for 15 min at room temperature.
    iv. Measure luminescence with a microplate reader (15 min after lysis).
    v. Also measure luminescence with a microplate reader at 30 min and 60 min after lysis, keeping the plate covered in foil in-between.
  c. Determine basal cytotoxicity normalized to total cell population.
    i. The second reading is taken at 3 time-points over 60 min to find steady-state luminescence signal. Use data from only 1 time-point to determine normalized cytotoxicity.
    ii. Normalize the cytotoxicity data by dividing the first reading (basal cytotoxicity per well) with the second reading (indicative of total cell population per well).
    iii. Express the data as a percentage.

### Cell death analysis in autophagy-deficient neurons by TUNEL assay

**Timing: 2 days**

To evaluate the impact of autophagy deficiency on neuronal viability at a single cell level, cell death analysis is performed using Click-iT Plus TUNEL Assay for In Situ Apoptosis Detection (Invitrogen). This assay is used to detect fragmented DNA in apoptotic cells by fluorescence signal.^22^^,^^23^ Following TUNEL staining, the cells are subjected to immunostaining with a neuronal marker such as TUJ1 in order to detect apoptosis specifically in neuronal cells generated from hESCs. The hESC-derived NPs are seeded into 24-well PO-L coated plates containing coverslips and then differentiated into neurons according to step 3. TUNEL^+^ apoptotic nuclei in TUJ1^+^ neurons are assessed by fluorescence microscopy after 3 weeks of neuronal differentiation where considerable cell death is observed in *ATG5*^*−/−*^ hESC-derived neurons^1^ (Figures 4B and 4C).

7. Perform TUNEL assay using Click-iT Plus TUNEL Assay for In Situ Apoptosis Detection, Alexa Fluor 488 dye (Invitrogen) on hESC-derived neurons, according to manufacturer’s protocol.
  a. Perform TUNEL Assay.
    i. Make up solutions from kit according to manufacturer’s instructions.
    ii. Remove media from each well of 24-well plate and wash once with 200 μL DPBS without shaking.
    iii. Fix cells on each coverslip with 200 μL of 4% paraformaldehyde (PFA) per well for 15 min at room temperature on a flat surface without shaking.
    iv. Slowly remove PFA and permeabilize cells with 200 μL of 0.25% Triton X-100 in DPBS per well, and incubate for 20 min at room temperature on a flat surface without shaking.
    v. Wash each coverslip slowly and gently twice with 200 μL deionized water (DNase/RNase free) per well for 5 min at room temperature without shaking.
    vi. Incubate each coverslip with 100 μL of TdT Reaction Buffer per well of 24-well plate for 10 min at 37°C in a humidified incubator.
    vii. Make up TdT reaction mixture according to manufacturer’s instructions.
    viii. Remove TdT Reaction Buffer slowly, gently add 50 μL TdT reaction mixture to each coverslip per well and incubate in a humidified incubator for 60 min at 37°C.
    ix. Wash each coverslip slowly and gently twice with 200 μL of 3% Bovine Serum Albumin (BSA) in DPBS per well for 5 min at room temperature without shaking.
    x. Make up fresh TUNEL Reaction Buffer Additive according to manufacturer’s instructions. Use this to make up TUNEL reaction cocktail according to manufacturer’s instructions.
    xi. Immediately add 50 μL TUNEL reaction cocktail to each coverslip per well slowly and carefully. Cover the plate in foil and incubate in a humidified incubator for 30 min at 37°C.
    xii. Gently remove the reaction cocktail, then wash each coverslip slowly and gently twice with 200 μL of 3% BSA in DPBS per well for 5 min at room temperature without shaking.
      ***Note:*** Take extra care at every step not to detach the fragile neuronal cells from the coverslip, even after fixation, by not shaking the plate. After TUNEL staining, protect the coverslips from light by covering the plate with foil.
  b. Perform immunostaining with TUJ1 antibody.
    i. Block each coverslip with 300 μL of 3% BSA in DPBS per well for 1 h at room temperature on a flat surface without shaking while covering the plate in foil.
    ii. Add 200 μL of TUJ1 antibody diluted in 3% BSA in DPBS (1:200) to each coverslip per well and incubate overnight at 4°C on a flat surface without shaking. Cover the plate in foil but do not put on a rocker or shaker.
    iii. Wash each coverslip slowly and gently twice with 200 μL of DPBS per well for 5 min at room temperature without shaking while covering the plate in foil.
    iv. Add 200 μL of Alexa Fluor 594 secondary antibody diluted in 3% BSA in DPBS (1:1000) to each coverslip per well and incubate for 1 h at room temperature on a flat surface without shaking. Cover the plate in foil but do not put on a rocker or shaker.
    v. Wash each coverslip slowly and gently twice with 200 μL of DPBS per well for 5 min at room temperature without shaking while covering the plate in foil.
    vi. Mount each coverslip onto a glass slide using ProLong Gold antifade reagent with DAPI. Incubate at room temperature in the dark for 15–30 min.
    vii. Seal coverslips onto glass slides using nail varnish, taking care not to move the coverslips on the surface of glass slides.
      **Pause point:** Imaging and analysis for TUNEL assay can be performed at a later date once coverslips are sealed and stored at 4°C. However, it is recommended to complete the analysis within a week so that the fluorescence does not fade.
  c. Analyze TUNEL^+^ apoptotic nuclei in TUJ1^+^ neurons by fluorescence microscopy.
    i. Image cells for TUNEL staining (green signal) and TUJ1 immunostaining (red signal) by fluorescence microscopy.
    ii. Assess the number of TUNEL^+^ apoptotic nuclei in TUJ1^+^ cells. Analyze ≥200 TUJ1^+^ cells per sample.
    iii. Calculate the percentage of TUNEL^+^ apoptotic nuclei in total number of TUJ1^+^ cells analyzed for each sample.

## Expected outcomes

Proper revival and maintenance of hESC-derived NPs can be observed by bright-field microscopy (Figure 1). Successful differentiation of hESC-derived NPs into neurons can be determined through morphological changes during the neuronal differentiation process by bright-field microscopy (Figure 2). Cellular identity of *ATG5*^*+/+*^ and *ATG5*^*−/−*^ hESC-derived NPs and neurons can be confirmed by the expression of cell-specific markers, such as NESTIN and PAX6 for NPs, and MAP2 and TUJ1 for neurons, via qPCR, immunofluorescence and immunoblotting.^1^

Autophagy deficiency, generated by knockout of *ATG5*, can be analyzed in *ATG5*^*−/−*^ hESC-derived NPs and neurons via immunoblotting by loss of ATG5–ATG12 conjugate, absence of LC3B-II (autophagosomes), and accumulation of p62 (autophagy substrate)^1^ (Figure 3). Additional analysis can be done via immunofluorescence or electron microscopy to detect lack of autophagosomes in *ATG5*^*−/−*^ cells, and by using lipid nanoparticle-mediated *ATG5* mRNA delivery to restore functional autophagic flux in *ATG5*^*−/−*^ cells.^1^

Basal autophagy is essential for cellular homeostasis and survival whereas loss of autophagy is detrimental to cells, particularly for post-mitotic cells like neurons.^1^^,^^6^^,^^7^^,^^20^^,^^24^ Autophagy dysfunction is reported in several neurodegenerative diseases that are considered to contribute to the disease pathology.^8^^,^^9^^,^^25^^,^^26^^,^^27^^,^^28^ In agreement with this, increased cell death can be observed in *ATG5*^*−/−*^ hESC- derived neurons via a cytotoxicity assay and TUNEL^+^ apoptotic nuclei in TUJ1^+^ neurons^1^ (Figures 4A–4C). Additional cell death analysis in *ATG5*^*−/−*^ hESC-derived NPs and neurons can be done via immunoblotting for cleaved caspase-3.^1^

## Limitations

This protocol produces a mixed population of forebrain neurons, typically comprising of glutamatergic and GABAergic neurons, following the withdrawal of growth factors after the NPs were generated from three-dimensional neurospheres using the dual-SMAD inhibition method.^3^^,^^4^^,^^10^^,^^11^ While morphological changes can be observed with brightfield microscopy, is not possible to confirm the cellular identity directly in experiments involving population level analysis, such as immunoblotting and microplate reader-based cytotoxicity assay. To work around this problem, it is possible to seed extra plates with or without coverslips to produce neurons specifically for the analysis of cell-specific markers via qPCR or immunofluorescence after neuronal differentiation. However, there can be some variation in the efficiency of neuronal differentiation between plates and the neuronal subtypes generated.

Another limitation is that the neurons become increasingly fragile as the differentiation progresses. It is possible to lose the cells through detachment or cell death during or after 3–4 weeks of neuronal differentiation, thus requiring starting the process again. Therefore, it is essential to take the greatest care at every step when handling the neuronal culture, and to keep a backup of each of the NP lines either in culture or as frozen stocks until the experiments involving neurons are completed.

## Troubleshooting

### Problem 1

The hESC-derived NPs are slow to replicate (step 2).

### Potential solution

Use early passage NPs that are cultured at high density to encourage expansion and prevent spontaneous differentiation. Add growth factors to the NP culture medium just before use (due to their instability when exposed to heat during warming the medium) and store the medium for a maximum of 1 week. Perform daily changes of NP culture medium to ensure a robust NP culture.

### Problem 2

The hESC-derived NPs have not differentiated properly (step 3) as cells continue to proliferate and/or retain NP morphology.

### Potential solution

Higher passage (>15 passages) NPs can undergo senescence that affects neuronal differentiation.^4^ Use early passage NPs to attain efficient generation of neurons. Also, maintain a backup culture of each NP line to re-seed for neuronal differentiation. Alternatively, revive frozen NP stocks, although time may be lost in re-establishing the culture.

### Problem 3

Neurons have died before maturation (step 3).

### Potential solution

Neurons are fragile in culture and have fewer contact points to the surface of the plate than NPs, thus making them vulnerable to detachment. It is important not to shake the plate or pipette the medium with too much force, otherwise the neuronal cells will detach from the plate surface and die. Also take care when moving cells in/out of the incubator and avoid closing the incubator door with force.

### Problem 4

Did not achieve separation of LC3B-I and LC3B-II bands, or LC3B bands lost from gel (step 5).

### Potential solution

LC3B-I and LC3B-II have low molecular weights, 18 kDa and 16 kDa, respectively.^14^ To achieve good separation of bands, use a 12% polyacrylamide gel.^18^ During SDS-PAGE, the dye front needs to have just left the bottom of the gel before stopping. If the gel is stopped too early the bands may not separate well, or if stopped too late the LC3B-II band may migrate out of the gel.

### Problem 5

Very few neurons are present on coverslips after the TUNEL assay (step 7).

### Potential solution

Neurons are extremely delicate even after fixation. During the TUNEL assay followed by immunofluorescence with neuronal marker, the neurons can detach and lift from the coverslips if pipetting with too much force. Take care to be gentle in pipetting at every step and do not use a shaker at any step.

## Resource availability

### Lead contact

Further information and requests for resources and reagents should be directed to and will be fulfilled by the lead contact, Sovan Sarkar (s.sarkar@bham.ac.uk).

### Materials availability

Further information and request for resources and reagents should be directed to the lead contact. The parental hESC line (WIBR3 hESC), originally generated and published by Rudolf Jaenisch lab at the Whitehead Institute for Biomedical Research, was used in this study by Sovan Sarkar lab at the University of Birmingham under material transfer agreements, UBMTA 15–0593 and 15–0595. We are glad to share the hESC lines generated in this study with reasonable compensation by requestor for its processing and shipping, and a completed materials transfer agreement.

### Data and code availability

This protocol does not involve or generate any specific dataset or code.
